# Provider perceptions of availability, accessibility, and adequacy of health and behavioral services for Latino immigrants in Philadelphia: a qualitative study

**DOI:** 10.1186/s12889-022-14066-z

**Published:** 2022-08-30

**Authors:** Ana P. Martinez-Donate, Nishita Dsouza, Sierra Cuellar, Gabrielle Connor, Claudia Zumaeta-Castillo, Mariana Lazo-Elizondo, Yoshiaki Yamasaki, Cristina Perez, Amy Carroll-Scott, Omar Martinez, Elizabeth McGhee Hassrick

**Affiliations:** 1 Department of Community Health and Prevention, Dornsife School of Public Health, Drexel University, Philadelphia, PA USA; 2 The Philadelphia AIDS Consortium, Philadelphia, PA USA; 3 WOAR Philadelphia Center Against Sexual Violence, Philadelphia, PA USA; 4 College of Public Health, Temple University, Philadelphia, PA USA; 5 A.J. Drexel Autism Institute, Drexel University, PA Philadelphia, USA

**Keywords:** Latino immigrants, Syndemic, Access, Availability, Quality, Health and behavioral services

## Abstract

**Objective:**

Latino populations in the United States are disproportionately affected by substance use, HIV/AIDS, violence, and mental health issues (SAVAME). A growing body of evidence demonstrates the syndemic nature of SAVAME and the need for integrated strategies to reduce their impact. This study sought to understand the network of SAVAME services for Latino immigrants in Philadelphia to inform future interventions for SAVAME prevention and mitigation.

**Methodology:**

Key informant interviews (*N* = 30) were conducted with providers working in Latino-serving organizations providing SAVAME services. Interviews were analyzed using thematic coding and grounded theory.

**Results:**

Latino-serving providers perceived a large need for, and important limitations in the availability, accessibility, and adequacy of SAVAME services for Latino immigrants. Gaps were seen as especially acute for mental health and substance use services, partly because of insufficient funding for these services. Latino immigrants’ lack of health insurance, immigration status, limited English proficiency (LEP), stigma surrounding SAVAME issues, and limited knowledge of available services were identified as significant barriers preventing access to services. Providers noted that scarcity of well-trained, culturally competent, and ethnically concordant providers reduced the adequacy of SAVAME services for Latino immigrant clients. The small size, low levels of infrastructure, and limited capacity were reported as additional factors limiting the ability of many Latino-serving organizations to adopt a syndemic approach in the prevention and treatment of SAVAME services.

**Conclusions:**

The results call for changes in the structure of funding streams and communitywide strategies to foster collaboration across SAVAME providers working with Latino immigrant clients.

**Supplementary Information:**

The online version contains supplementary material available at 10.1186/s12889-022-14066-z.

## Introduction

Latino populations in the United States are disproportionately affected by co-occurring substance use, HIV/AIDS, violence, and mental health issues (SAVAME) [1]. Although these issues are often examined and addressed independently, a growing body of evidence demonstrates the synergistic nature of these issues and the need for integrated strategies to reduce their impact. For example, substance use is associated with higher HIV transmission [2], poor mental health [3], and violence victimization [4]. Reciprocally, untreated mental health issues are associated with increased HIV risk behavior [5], violence [6] and substance use [7]. Domestic violence (DV), a highly documented form of violence victimization in this population [6], is a risk factor for HIV infection [8]. DV is also a known cause of stress and trauma in this population and often goes untreated, resulting in a need for mental health services [6].

Syndemic theory posits that certain health problems “interact synergistically” within a community and stem from common social determinants, such as immigration status and poverty [9]. SAVAME in the U.S. follows this pattern, as these health issues tend to cluster and affect disproportionately populations who experience poor physical and social conditions [1]. Examining the interactions of SAVAME through a syndemic lens identifies significant shared upstream factors that drive these health disparities for Latino populations, such as poverty, lack of health insurance, migration, discrimination, and residential segregation [9]. Recently, these factors have been exacerbated by the COVID-19 pandemic, which has led to economic shocks and disruptions in the provision of needed health and/or social services. Following mandatory lockdowns, employment among Latinos remained 7.2% lower than pre-pandemic levels, the largest loss among all racial and ethnic populations [10].

Evidence on best strategies to address the SAVAME syndemic among Latino immigrants is sorely needed. A syndemic approach calls for identifying risk and protective factors connecting these conditions and developing integrated interventions to simultaneously address them [11]. Greater integration of SAVAME prevention and treatment services for Latino immigrants is critical to reduce the impact of the syndemic on this group [12, 13]. Identifying SAVAME services and community resources available to Latino immigrants and the extent to which they are accessible, adequate, and integrated across SAVAME issues represents a first step to that end. This qualitative study is part of a community academic collaboration to better understand the strengths and gaps of the network of SAVAME services available to Latino immigrants in Philadelphia, a large city in the mid-Atlantic region of the United States, with the ultimate goal of informing future interventions for SAVAME prevention and mitigation.

Philadelphia has a sizable (15.2% of overall population) [14], diverse and rapidly growing Latino community. Among them, one in five is foreign born immigrants [15], while an estimated 25% of the foreign-born residents is unauthorized [16]. The city includes two primary ethnic enclaves: one in the North, with a larger proportion of (predominantly Puerto Rican origin) Latino residents, and one in the South and Southwest Philadelphia, with a smaller but fast-growing (predominantly of Mexican origin) Latino community [17]. The Latino population is also becoming more heterogenous in its ethnic diversity due to growing numbers of Mexican, Central, and South Americans. Latinos in Central and South Philadelphia are susceptible to rapid urban development in the city, which is displacing populations further out of the city core into areas where the housing stock is more available and affordable, such as the Northeast city area or the suburban metropolitan region [18]. In addition to anecdotal testimonials, there is much evidence of the SAVAME syndemic present in the city’s Latino population. Compared to other racial and ethnic groups, Latinos in this city have the highest rate of new mental health diagnoses and the second highest rate of new HIV diagnoses, binge drinking, and opioid-related mortality [19].

As in other parts of the U.S., the supply of Latino-serving providers in Philadelphia has grown to try to meet the demand for services created by a Latino population who has nearly tripled in size since the year 2000. However, disparities in SAVAME for Latinos in the city and anecdotal accounts of the limited resources to address the syndemic in this population persist. Recent studies show that geographic and service sylos hinder interorganizational collaboration, a critical element to more effectively address the SAVAME syndemic [20].

This study is part of a larger, mixed-methods formative assessment in Philadelphia, Pennsylvania to inform intervention strategies to reduce the disproportionate impact of SAVAME on Latino immigrant communities. Among other components, the larger project included mapping and surveying Latino-serving organizations in the city and conducting key informant interviews (KII) with providers in these organizations. This study presents the findings from the KIIs, which were informed by findings from the aforementioned survey of organizations, The LatIno NetworK of Services (LINKS) Survey, conducted in 2018–2019. This survey’s sampling framework was a roster of 43 organizations identified by our community academic team as providers of SAVAME-related health, educational, legal, or social services to adult Latino immigrants (e.g. – Mexicans, Central Americans, South Americans) or U.S. citizens from Puerto Rico living in the city. The survey (*N* = 31, 72% response rate) showed that syndemic services for Latino immigrants in Philadelphia ranked low in terms of availability, accessibility, and adequacy [21]. These three factors are considered important dimensions within the general concept of access to services [22]. Mental health providers perceived less access to their services than HIV/AIDS, domestic violence, and/or substance use providers. Study findings also pointed to the necessity for increased training and/or technical assistance to provide services adequate for Latino culture, language, and values. In addition, the survey showed some level of referral collaborations between organizations providing complementary SAVAME services, but limited administrative or planning collaborations between them [20]. The aim of this qualitative study was to supplement LINKS quantitative findings about the network of Latino-serving organizations in the community and provide a more in-depth characterization of the availability, accessibility, and adequacy of SAVAME services for Latino immigrants in Philadelphia, as perceived by staff and providers who serve this population, and to inform future interventions for SAVAME prevention and mitigation.

## Materials and methods

### Study design and procedures

A cross-sectional qualitative study was conducted. Thirty participants were recruited to participate in key informant interviews between April 2019 and June 2020. Organizations included in the LINKS roster were selected using purposive sampling, to cover different SAVAME services and geographic locations within the city. A research team member contacted the selected organizations by phone or email to identify a staff member who was familiar with the organization’s services for Latino immigrants and could participate in the study on behalf of the organization. To be eligible, key informants had to be 18 and older, working for at least 1 year in the organization included in the LINKS roster, and be a provider or supervisor of SAVAME services in said organization. Eligible individuals were asked to provide informed consent and take part in a semi-structured interview.

Interviews took place in a private meeting room either in person at the first author’s academic institution or at the key informant’s service location, over the phone, or Zoom (added as an option due to the COVID-19 pandemic). All interviewers were administered by a trained research assistant using a semi-structured interview guide developed by the authors, which included questions related to the key informant’s organization, the need for SAVAME services in the Latino immigrant community, the availability, accessibility, and adequacy of services provided to the Latino immigrant community, and collaborations with other Latino-serving organizations. Questions were informed by previous research and findings from the LINKS quantitative survey [21]. All interviews except for one were conducted in English; one was conducted in Spanish as per the key informant’s preference. Key informants were also asked to complete a brief survey on demographics and organizational characteristics. Interviews lasted approximately one hour, and each key informant received a $30 gift card for their participation. The community was engaged in multiple ways and along each phase of this research. Community partners were involved in the purpose and design of the study, writing of the grant proposal, review of the interview guides, and selection of key informants. Partners also helped interpret the findings. In addition, key findings from the qualitative interviews were presented at a town hall of the Philadelphia Latino Health Collective and key informants were invited to attend the presentation to validate the findings and their interpretation. All study activities were approved by Drexel University Institutional Review Board (IRB).

### Sample characteristics

Key informants included 21 females and 9 males, with a median age of 35–44 years (see Table 1). Most (60%) had completed graduate studies and two-thirds identified as Hispanic or Latino (67%). Key informants represented 9 different countries of birth. The largest group was that of participants were born in the U.S. mainland (45%), followed by Mexico (17%) and Peru (10%). Almost a quarter (23.3%) supervised substance use treatment programs, 20.0% oversaw programs involving HIV/AIDS treatment and/or prevention, 23.3% supervised domestic violence or interpersonal violence programs, 46.7% oversaw mental health programs; and 36.7% provided or supervised other non-SAVAME specific social services. Role types varied, with most providers engaged in administrative work (76.7%), direct service provision (36.7%), and in a leadership capacity (50.0%) within the organization.

**Table 1:** Demographics of key informant Latino-serving providers in Philadelphia, 2019–2020 (*N* = 30) ^a, b^

	**n**	**%**
Female gender	21	70.0
Age (years)		
Less than 35	11	36.7
35–44	9	30.0
45–54	2	6.7
55 or over	8	26.6
Education level		
Master’s degree or higher	18	60.0
Bachelor’s degree	9	30.0
Did not complete college	3	10.0
Self-reported racial identity^c^		
White	12	41.4
Black	2	6.9
Indigenous of the Americas	7	24.1
Other / Multiracial / Prefer not to answer	8	27.5
Hispanic/Latino ethnicity	20	66.7
Country of birth		
USA Mainland	13	44.8
Puerto Rico	2	6.9
Mexico	5	17.2
Peru	3	10.3
Other Latin American countries Dominican Republic	6	20.5
Bilingual English/Spanish	27	90.0
SAVAME services provided/overseen ^c^		
Substance Use	7	23.3
HIV/AIDS	6	20.0
Domestic Violence	7	23.3
Mental Health	14	46.7
Type of role ^c, d^		
Direct service provider	11	36.7
Leadership role	15	50.0
Administrative work	23	76.7
Length of time in current role		
Two or less	9	32.2
3–5 years	8	28.6
6–10 years	5	17.9
10 + years	6	21.4

^a^ Percentages were calculated excluding missing data

^b^ Categories not shown had zero frequency

^c^ Categories are not mutually exclusive

^d^ Question not asked in demographic survey; responses were abstracted by research assistants from interviews

**Table 2:** Demographics and organizational characteristics of key informant Latino serving providers in Philadelphia, 2019–2020 (*N* = 30) ^a,b^

	**n**	**%**
Primary location of service provider		
North Philadelphia	15	50.0
South or Center Philadelphia	15	50.0
Percent of clients who are Latino immigrants		
More than 80%	9	32.1
50% to 80%	10	35.7
Less than 50% / Don’t know	9	32.7
Bilingual Spanish staff at their organization		
All	17	60.7
Most	8	28.6
Some/Few	3	10.7
Services for which translation is available		
All	15	55.6
Most	4	14.8
Few/None	5	18.5
Don’t know / Prefer not to answer	2	7.4
Not applicable (staff is bilingual)	1	3.7
Staff/providers are offered cultural competency training		
Often	17	58.6
Sometimes	9	31.0
Rarely	2	6.9
Never	1	3.4
Organization hires bilingual (Spanish-speaking) staff/providers		
Often	24	82.8
Sometimes	5	17.2
Organization uses immigrant-friendly intake procedures^1^		
Often	23	79.3
Sometimes	4	13.8
Rarely	1	3.4
Don’t know / Prefer not to answer	1	3.4
Organization collaborates with other organizations that offer complementing services		
Often	21	72.4
Sometimes	7	24.1
Rarely	1	3.4
Organization assesses unmet needs among Latino immigrant community		
Often	10	34.5
Sometimes	11	37.9
Rarely	6	20.7
Never	1	3.4
Don’t know / Prefer not to answer	1	3.4
Organization conducts outreach to increase awareness of services		
Often	16	55.2
Sometimes	7	24.1
Rarely	3	10.3
Don’t know / Prefer not to answer	3	10.3
Perceived level of integration of SAVAME services in Philadelphia		
Excellent	2	8.3
Very Good	1	4.2
Good	4	16.7
Fair	5	20.8
Poor	8	33.3
Don’t know / Prefer not to answer	4	16.7

^a^ Percentages were calculated excluding missing data

^b^ Categories not shown had zero frequency

^c^ Categories are not mutually exclusive

^d^ Question not asked in demographic survey; responses were abstracted by research assistants from interviews

### Analytical strategy

All interviews were audio recorded and transcribed verbatim using an external transcription service. Transcriptions were reviewed by an additional team member for quality purposes. The Spanish interviewed was transcribed and translated to English by a bilingual team member and reviewed by a second team member. To prepare for coding, interview transcripts were cleaned, summarized, and anonymized. Interviews were analyzed using thematic coding and grounded theory [23]. A mixed inductive and deductive coding approach was implemented. The research team developed a codebook with a priori codes based on the interview guide, the main research questions, the reading of the first few transcripts, and previous research. The codebook was expanded during the coding process to reflect emergent inductive codes from the data. The codebook was uploaded to NVivo 12 (QSR International, Melbourne, Australia) and clean transcriptions were coded by three of the authors. Coders also utilized analytic memos to identify overarching themes among key informant interviews.

To ensure homogeneous use of each code across coders, the first 2 interviews were coded as a group and 6 additional interviews selected at random were coded by two independent coders. Inter-rater reliability between pairs of coders was calculated bi-weekly during the coding process. The inter-rater reliability measured the degree of agreement between coders on each interview that was coded multiple times. Inter-rater reliability ranged from 91.46 to 94.72, indicating a high level of agreement between pairs of coders. Discrepancies and errors were identified and resolved by group consensus. Codes were combined into salient themes and subthemes and included in analytical matrices along with interview excerpts illustrating them. The results were iteratively reviewed, interpreted, and vetted by the community academic research team during several work meetings. This paper presents themes and subthemes related to the need for, availability, accessibility, adequacy, and integration of SAVAME services for Latino immigrants in the local community.

Demographic and organizational survey data were analyzed using descriptive statistics and the Statistical Package for the Social Sciences (SPSS) software, version 25.0 (IRB, Armonk, NY).

## Results

### Organizational characteristics

Half of organizations’ primary location was in North Philadelphia while the other half were in South or Center Philadelphia. Based on survey responses, Latinos represented *half or more* of the client population served for nineteen (69%) of the organizations. Sixty-one percent of the organizations had *all* bilingual staff, 83% hired bilingual (English/Spanish) staff *often*, and 56% offered translation for *all of their services*. The majority of the organizations (79%) offered immigrant-friendly intake procedures *often,* but only 59% provided cultural competency training *often*. Collaboration with organizations that offered complementary services was described as happening *often* for 72% of organizations. Only 34% of the organizations assessed unmet needs among Latino immigrant clients *often* while 55% conducted outreach to increase awareness of services *often*. The percentage of key informants who rated integration of SAVAME services in the city as *excellent or very good* was only 12% (Table 2).

**Fig. 1:**
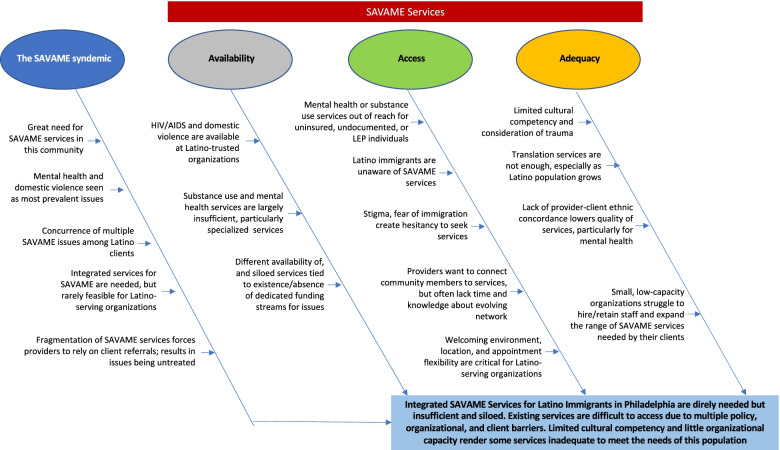
Summary of perspectives of Latino-serving providers on Latino immigrants’ access to SAVAME services in Philadelphia, 2019–2020 (*N* = 30)

Figure 1 presents a summary of the main themes emerging from the qualitative analyses of the interview transcripts by main area of analyses.

### Need for SAVAME services

Participants perceived a large need for SAVAME services for Latino communities in Philadelphia, emphasizing the impact of mental health and domestic violence. The need for services in these two areas was tied to frequent experiences of trauma among Latino immigrants prior to leaving their home countries, during their journey to the U.S., and/or after reaching their destination in the U.S. Providers noted that many immigrants endure abusive domestic relationships and live in high-crime areas, which increase the risk for violence victimization.

*“I know that there's a lot of folks who experience domestic violence, who have come here through human trafficking from another country. When they get here, they're still in some sort of abusive relationship or abusive system.” (KII #22)*

Staff at these Latino-serving organizations was also aware of the connectedness of SAVAME issues and, often struggled with how to respond to their clients’ SAVAME-related needs in areas not covered by their organization. One female staff at a mental health clinic commented that many of her Latino clients have *“co-occurring disorders with substance abuse.”* Despite the concurrent nature of these syndemic factors and providers’ recognition of the need for integration of services, few organizations were able to provide comprehensive SAVAME services and most were able to work in only one or two SAVAME issues. Some providers described successful efforts at their organizations to expand the range of services to address additional SAVAME issues and meet more of their clients’ needs within their organization.

“So, people that access [PROVIDER’S ORGANIZATION] usually do not want to access services elsewhere and that's kind of how we ended up with adding so many services” (KII #13)

However, the perception of many providers was that their ability to offer services outside their main SAVAME area of specialization was insufficient to meet the needs of their Latino immigrant clients. When services were not available at their organization, providers reported they suggest alternatives or refer clients to other agencies in hopes they could meet the needs of their clients.

*“If their need as a substance user is so great that we think they do better in another setting, we'll [refer them to another provider]. We don't do methadone treatment, we don't do suboxone treatment and we don't have an intensive outpatient program.”(KII #0)*

The importance of integrating SAVAME services in culturally competent and well-trusted organizations was emphasized by the study participants. For example, some noted that there are times when clients with SAVAME issues would come to their organizations to obtain help, even when the needed services are not offered there, due to other characteristics that render these places well-trusted among the Latino immigrant population (e.g.friendlier, Spanish-speaking providers, availability of other useful services). This behavior increased the risk of leaving those SAVAME issues untreated or treated only with less specialized services than necessary.

“[For substance use], we refer them [to specialized substance use services] but some of them keep coming.” (KII #12)

### Availability of SAVAME services

Providers communicated that availability of services for Latino immigrants in the city varied across SAVAME factors. Resources related to HIV/AIDS and violence victimization were described as generally more available compared to substance use and mental health services. Providers reported that, with minimal exceptions, services for HIV/AIDS were well integrated into larger health service providing organizations (see Table 3).

“I think HIV and AIDS, there are a lot of organizations that provide those services, and domestic violence.” (KII #22)

**Table 3:** Main themes and illustrative quotes reflecting providers’ perspectives on availability of SAVAME services for Latino immigrants of Latino-serving providers in Philadelphia, 2019–2020 (*N* = 30)

Perspectives on Availability of SAVAME Services	
HIV/AIDS and domestic violence described as more available at Latino-trusted organizations	“HIV has many programs targeting [the Latino community]. For example, [NAME OF ORGANIZATION] has three or four programs targeting [Latinos] where they go and they eat, they have activities, they have events. We prefer to refer our clients to them because they have support groups, they have family group, they have individual therapy. They have activities in the community, outside.” (KII #12)
Substance use and mental health services clearly insufficient, particularly specialized services	“Every psychiatrist schedule is packed and there are always people hoping for it, you know, and they're only seeing them for 10 min. The bottleneck is getting to see the psychiatrist. You can get an intake right away. You can see a therapist within the week. It's the psychiatrist that takes time… and, all that's true of all of these [mental health] clinics.” (KII #10) “I would say that for substance use there are not [services]. There is an inpatient program that targets Latinos. But other than that, it is not rare to go into like a [medication-assisted treatment] clinic and no one there speak Spanish.” (KII #13)
Availability dependent on dedicated funding streams for issues (e.g. Ryan White Act, Victims of Crime Act)	"The number one problem for the poor in the United States… is housing. And it's the one time HIV is really good news. If you come in and say I'm homeless, and I have AIDS, I go, “Oh thank God,” because I can get you into an apartment in a month if you have AIDS. And that's been in place since the time of the Ryan White grants in the 1980s." (KII #0)
No similar funding streams for substance use or mental health	“We're lucky to have some funding to help support a mental health at [NAME OF ORGANIZATION] so we have both a psychologist and a therapist social worker who see patients for mental health stuff… But you know, that's only, that's like two nights a week that we have that. So, it's not enough for everybody, of course. (KII #8)

Study participants also remarked that, compared to mental health and substance use services, HIV/AIDS and domestic violence services were more frequently offered by well-trusted Latino-serving organizations and in culturally competent ways. This perception was often correlated with greater readiness and willingness to refer clients to these organizations. In contrast, culturally appropriate mental health and substance use programs for Latino immigrants were viewed as particularly lacking in the city.

*"I think out of all [SAVAME factors], mental health and substance abuse are the ones that are struggling the most [with being adequate for Latino populations in Philadelphia]. I think HIV and AIDS, there are a lot of organizations that provide those services, and domestic violence. The one where I think we feel the impact is, is mental health and substance abuse services and just connecting people to that resource because the folks who are accessing domestic violence and HIV and AIDS services are the ones who also need mental health services as well. And it can be challenging to get them into that.”(KII #22)*

The limited availability of mental health services was seen as especially acute for psychiatric services. While some organizations reported to be able to provide intake and basic services, providers reported that specialized care and psychiatric services were much harder to find for Spanish-speaking populations in the city. Similarly, culturally competent substance use services for the Latino immigrant community were regarded as direly needed, but clearly insufficient. KII#5 lamented that for substance abuse *“it is really difficult to find treatment for this population.”* Study participants also noted that scarcity of substance use services was especially acute for higher-order, specialized substance use services, such as medication-assistance treatment, and also expressed challenges with referring clients or patients to these services due to cost, language, or insurance barriers. As a result of this shortage of services, many providers described mental and behavioral health as largely under addressed issues among Latinos in Philadelphia.

Funding is a key determinant of services being available and, unfortunately, funding streams are often siloed to supporting one service area while other areas lack necessary resources. In the case of HIV/AIDS services, participants attributed the existence of more resources available to Latino immigrants to the existence of the Ryan White Act (Doshi, 2015).

"[For] *HIV services, you have Ryan White funding. So, basically, all the organizations that receive Ryan White can provide services to people without insurance... but basically for HIV, there’s resources for immigrants, I will say so." (KII #21)*

For our key informants, the existence of this HIV-specific funding stream made it easier to provide wraparound services and to address critical social and cultural determinants of health, such as housing or stigma, for Latino immigrants living with HIV.

*“We offer testing and […] we specifically created a program and then combated HIV stigma in Latino communities because we can have the service and it can be available to anyone, but if we're not addressing why people aren't coming through the door to get tested… and for us, we know that a lot of it has to do with stigma.” (KII #25)*

For domestic violence, providers also acknowledged the important role of specific funding programs, such as the Victims of Crime Act (VOCA), that facilitate the provision of services, including mental health services, for Latino clients who are victims of any type of crime, regardless of immigration status [24].

*“Previously our domestic violence funding came for working with Spanish or like Latino survivors Latina or Latino survivors of domestic violence. But then we switched to VOCA, which is the Victims of Crime Act, and that funding is way more general and can apply to anyone so that's why our client base, I suppose, for my particular program is changing. VOCA is just... They just have a lot of funding. I think it's a good and very consistent source [of funding].” (KII #14)*

In contrast with these two specific and large funding programs for HIV/AIDS and victims of violence, no similar dedicated funding streams for mental health or substance use were mentioned by our KIIs. In fact, providers complained about the limited funding available to support these types of services within their organizations.

“A lot of times it’s very difficult for us to treat Latino populations in regards to substance abuse because we just don't have the staff, you know.” (KII #15)

### Accessibility of SAVAME services

Providers identified different types of barriers impeding access to existing SAVAME services in the city. First, some study participants recognized structural and language barriers, especially poverty, lack of insurance, documentation status, and limited English proficiency. For example, the gap in mental health services was not seen by study participants as unique for Latino immigrants, but rather as a limitation extending to poor and uninsured individuals in general. Low levels of health insurance and low income were noted as chief reasons mental health services are out of reach for Latino immigrants in the city (see Table 4).

*“For immigrants particularly, there’s basically no services for mental health. If you have no insurance, you're depending on the client to be able to afford to cover the cost and a lot of people can’t. So, organizations like ours, [we] don’t advertise our services. And we have 400 clients. And we don’t advertise, we don’t send flyers, we don’t do anything... They’re seeking from us because there are no [other] services for mental health." (KII #21)”*

**Table 4:** Main themes and illustrative quotes reflecting Latino-serving providers’ perspectives on accessibility of SAVAME services for Latino immigrants in Philadelphia, 2019–2020 (*N* = 30)

Perspectives On Accessibility of SAVAME Services	
Structural barriers: Poverty, lack of health insurance, documentation, and language barriers	*“[Accessing] substance abuse [services] is impossible. It's really impossible […] people using drugs or stuff like that, it is impossible to find a place where they can go because of legal documentation or because the places don’t take them seriously or like language access.”(KII #19)*
Organizational factors: Welcoming environment, location, and appointment flexibility are critical for Latino-serving organizations	*“There are people who are actively using drugs that don’t have anywhere to live and they don't really feel welcome in a lot of spaces, but they always knew they could come to [NAME OF ORGANIZATION]. We see most people like every other day or every day. People that access [NAME OF ORGANIZATION] usually do not want to access services elsewhere and they don't want to leave the Kensington area. […] the shelter is so far away and there's no transportation and if you are using drugs, you're going to wake up sick and you have no way to get back to Kensington.” (KII #13)*
Providers: Key connectors, but lack time and knowledge about available services	*“I do find myself sometimes like googling certain things cause I’m like, okay, [the clients] definitely need to be referred somewhere, but I just don’t know what or if it’s available. So, I’m just sitting there trying to find anything. I didn't really, like get taught or anything of, like, specific Latino organizations […] It's kind of frustrating because I'm like, ‘I should know these things’ and maybe if it was a more English-speaking patient that I'd be like, ‘oh, okay, just go here and go there.’” (KII #17)*
Latino immigrants: Unaware of services and fearful of consequences of seeking services	*“I think once they are connected to the [DOMESTIC VIOLENCE] services, it's very easy, but making sure that they're aware of the availability of those services and that they are not afraid of any type of like ICE involvement or anything like that. […] I think it's just really getting the word out about the availability of the services and the safety of the services." (KII #22)*

In addition, several providers stated that some organizations providing SAVAME services, including their own, require that individuals provide documentation and be insured to receive services. In addition, regardless of actual requirements, many Latino immigrants in Philadelphia assumed they need documentation or insurance and, as a result, did not even seek out needed care.

*“So, when I'm working with clients that […] are not eligible for any kind of public benefits or health insurance like that, it feels a lot trickier to find services both for mental health and physical health” (KII #29)*

As such, providers stated that meeting the more fundamental needs of the Latino immigrant population in Philadelphia required the provision of legal and financial assistance to overcome these barriers and secure some of these pre-requisites. Ideas mentioned included helping eligible Latino immigrants apply for Medicaid, legal status, and work authorization.

Providers also shared that for many Latinos, the lack of access to mental health and substance use services was further aggravated by language barriers and the low number of organizations that offered these services in Spanish.

“…and I think that [mental health] need is very great, and access is very difficult especially if you don’t have insurance and also if you don’t speak English.” (KII #8)

In addition, when discussing domestic violence services, our key informants discussed how accessing services and reporting violence victimization to law enforcement can be challenging for Latino immigrants due to stigma, fear of repercussion from an abuser, and apprehension about the potential to alert immigration officials. Along these lines, providers also emphasized the need to publicize the availability and the safety of these services in order to increase access among Latino immigrants.

*“As far as the violence issue, I think there's a lot of fear about that part of why [immigrants] are targets […] they're afraid of going to law enforcement or reporting things because of lack of documentation. And they just kind of feel like there's not resources available to them." (KII #8)*

At the organizational level, our key informants identified a welcoming environment, convenient location and appointment flexibility as critical elements of an organization’s operations to help make services more accessible for Latino immigrants. Providers reflected on their unique position to identify their clients’ needs and to help them connect to right services. They recounted trying to increase awareness about other services among their Latino immigrant clients. They felt responsible for promoting their own services, so they become better known among Latino-serving providers and community members.

*“…you're the entry level for them to be able to access as many different services as possible. So even though they may be coming for one thing it's your job to identify what other resources they can benefit from and try to help them connect as best as possible… because that may be the one and only time that [this] individual comes to a place like this for services so they may not have that opportunity to identify them again without our help” (KII #16)*

However, study participants conceded that they had insufficient knowledge, training, and time to keep abreast of services available in the community and these barriers jeopardized their ability to serve as connectors for these clients. Providers reported being unsure about the range of SAVAME-related services available and accessible to their clients, partly because of the evolving nature of the network of organizations and services.

Limited awareness about services, low self-efficacy regarding how to navigate the system, and insufficient social and digital skills among Latino immigrant community members were also noted as important obstacles for access to SAVAME services in the city. Because of these issues, providers emphasized the need for organizations that provide SAVAME services to engage in person-to-person outreach and education efforts in heavily Latino neighborhoods, so community members learn about these services.

*“…a lot of folks that are migrating here don't know the services, don't know what the public health department is, don't know how to access these databases or these like services. And so, a lot of it is word of mouth. […]... Like we, being a Latino organization, we purposely go to these neighborhoods that are predominately Latino. But I don't think a lot of other agencies do the same. And a lot of them kind of expect folks to already walk through the doors. So, I think just, if they can increase their efforts to just be known in the Latino community that you can receive services here.” (KII #15)*

### Adequacy of SAVAME services

Key informants reported that SAVAME services for Latino immigrants and other disadvantaged populations were in many cases of lower quality than those available to native and more privileged populations. This was particularly noted for mental health services. As a result, providers stated they had low level of trust and often hesitated to refer their clients to some mental health services in the city (see Table 5).

*" Outpatient mental health treatment for poor people is often not great and if you speak Spanish, it's horrific. We won't even refer [clients] to some of the places. That would be good to improve upon. Not only increase but improve upon the quality of what people in poverty are able to get." (KII #7)*

**Table 5:** Main themes and illustrative quotes reflecting Latino-serving providers’ perspectives on adequacy of SAVAME services for Latino immigrants in Philadelphia, 2019–2020 (*N* = 30)

Perspectives On Adequacy of Services	
Limited cultural competency and tailored trauma-informed services	“A lot of organizations are not culturally competent to serve Latinos […] at this point we have such a large Latino population that it should be everyone's main focus to have, kind of like, to make it a point to-to know how to serve Latinos. […] I think having bicultural providers and having people just be aware of the issues that people are facing outside of their health and outside of HIV and how that could affect them, like [as a] whole person and how [these] can make it difficult for them to address these different issues.” (KII #13)
Shortage of ethnically concordant providers	“…or no one there, like they don't have… what’s a [politically correct] way to say this? They don't have like a staff that looks like the population that they're serving you know, which makes it really hard for people to connect with them even if they do speak Spanish.” (KII #13)
Need for translation services reduces quality of provider/client encounter	“It's not fair to people that someone else that is not a medical provider is in the room with them translating back and forth between them […] and in those interactions there's a lot of things lost. Especially because I know most people will use like the phone line which I've seen in action and it is terrible. It's like it just doesn't work and it's just awkward.” (KII #13)
Low-capacity organizations face challenges to hire, train, retain staff necessary to increase quality or expand services	“Within Latinx-specific organizations, I think that [services are] adequate only if they have enough people on staff to help. That's the main thing. […] In [NAME OF ORGANIZATION], there’s only three people on staff and […] I work part time. So, it's like, if you have a lot of staff that works part time, it's like juggling the internal organizational things in addition to servicing people […] …and we want to help more but we can't help because we don't have capacity to. […] Like as a small org, it gets really hard. I know that we want to do so much more, but it's just like we would fail at it all.” (KII #27)

When inquired about the reasons for low levels of adequacy, providers criticized the limited cultural competency and the low number of bicultural staff at health and social service organizations in the city. They indicated that consideration of cultural norms and structure in service delivery was currently lacking and interfered with the ability to meet the needs of Latino immigrant clients. Some providers mentioned that cultural competency must go beyond having translation services or providing services in Spanish and ideally should entail providers that are ethnically concordant with their Latino immigrant clients.

*“Especially for my community, the Mexican community, there are not enough Mexican psychologists or Mexican therapists. I feel more comfortable speaking to a Mexican psychologist who is originally from Mexico who speaks the language and understands my culture and, you know, the way I may think and I was raised compared to a U.S. psychologist who may speak Spanish fluently but who didn’t have contact with my culture.” (KII #20)*

Beyond cultural and linguistic competency, one key informant remarked on the need to enhance the general professional expertise, knowledge base, and skill set of mental health providers currently serving this population. This informant complained that due to the small pool of bilingual mental health professionals available in the area, some organizations end up hiring providers who may have a degree in a health-related profession but lack adequate training in psychology, social work, or counseling. He noted “the fact that you have a health-related profession, maybe, does not mean you are trained in mental health counseling. Moreover, even if you are a mental health provider it does not make you a professional. The problem goes deeper than lack of available SAVAME resources but it’s also about the quality of the ones that do exist” (KII #21).

Several key informants also discussed trauma, as an additional factor that must be considered when serving Latino immigrant clients. According to some providers, traumatic experiences before, during, and after migrating to the U.S. are the reason why many Latino immigrants need SAVAME services and why these services must be trauma informed. Providers criticized that, except for some agencies whose focus was primarily Latino populations, staff in many of organizations offering SAVAME services had limited awareness of the traumatic past many immigrants carry or the dire circumstances in which they live once they reach the U.S. In their view, insufficient cultural competency and trauma-informed practices hindered the ability of many organizations to meet adequately the SAVAME needs of their Latino immigrant clients.

*“…and I think there's not enough training around [trauma], there's not enough conversation about that… especially all the groups that are interested to work with this population and to work around that area, to put serious time and effort and resources into learning how to deal with this type of trauma.” (KII #19)*

An additional factor mentioned by providers was the limited capacity of most Latino-serving organizations in the city, along with high caseloads and staff turnover. With some exceptions, many providers talked about their organizations being small and suffering from large economic constrains that made it difficult for them to hire, train, and retain as many bilingual and bicultural staff as they would need to serve more Latino immigrant clients and to offer the breadth of services that they see these clients need. Lack of capacity also reduced opportunities to apply for funding necessary to develop the infrastructure needed to expand their services, in a cycle that perpetuated these organizations remaining small and at a low capacity.

## Discussion

This qualitative study aimed at characterizing the need for, availability, accessibility, and adequacy of SAVAME services for Latino immigrants in Philadelphia, as perceived by Latino-serving providers working with this population. Using key informant interviews, we built on previous findings from LINKs, a quantitative survey of Latino-serving organizations previously conducted by our team, and set out to dive deeper into this area of inquiry. The findings from this qualitative study underscore the need for SAVAME services among Latino immigrant communities, especially domestic violence and mental health, and the syndemic nature of these health and behavioral issues. Providers’ responses reflected the concurrence of several SAVAME factors in many of their clients (e.g. substance use and mental health) and the need for better service integration and coordination to address these syndemic issues. These findings are consistent with previous literature describing the SAVAME syndemic in Latino populations in the U.S. [1, 25] and earlier calls for a syndemic approach in the prevention and treatment of these interrelated epidemics [26]. The providers’ responses were also in line with findings from the earlier LINKS survey, which showed that only 20% of the Latino-serving providers surveyed considered integration of SAVAME services in the city was good and 19% reported that their organizations routinely screened their clients for all four SAVAME factors [21]. Together, these results highlight a missed opportunity to identify and address concurrent SAVAME needs experienced by Latino immigrants presenting at Latino-serving organizations.

When considering the availability of services for each of the syndemic issues, there is more confidence among providers on the sufficiency of resources for HIV/AIDS and domestic violence, due to relatively stable federal funding streams supporting services in those areas. This generous funding programs make it possible for trustworthy organizations to develop and maintain culturally competent programs to cover the needs of Latino immigrants living with HIV or subject to violence victimization. In contrast, there is concern among providers about the low availability of mental health and substance use services for poor and uninsured individuals, among whom Latino immigrants are overrepresented. The limited funding creates important challenges for Latino-serving organizations to hire, train and retain bilingual and bicultural staff to support Spanish-language, culturally tailored, trauma-informed substance use and mental health services for immigrant communities. The results resonate with and expand the LINKS data, which also showed lower availability scores for mental health and substance use services in Philadelphia compared to HIV/AIDS and domestic violence [21].

Our findings also highlight multiple, compounding barriers Latino immigrants face to access existing SAVAME services. Lack of health insurance, low income, limited English proficiency, and irregular immigration status make some of the existing services inaccessible for this community. In addition, Latino immigrants’ lack of knowledge regarding available SAVAME services, stigma surrounding these syndemic issues, and fear of deportation create hesitancy to seek services and further reduce access to SAVAME services. Organizational factors, including the scarcity of well-trained Spanish-speaking, culturally competent, ethnically concordant providers, as well as the limited use of trauma-informed approaches and low capacity of many SAVAME service organizations lessen the quality of the services offered, especially mental health and substance use, and contribute to these issues not being effectively treated in this population. These findings are consistent with previous studies exploring the perceptions of providers on availability of health and social services for immigrants and refugees in other U.S. regions, which have found that geographic accessibility, language and cultural barriers, cost and insurance coverage, and policies related to immigration status impacted access to services among these populations [27–29]. Some of the barriers documented by our study are likely to apply to other immigrant populations. Asian immigrants, who represent a large segment of the foreign-born population in Philadelphia, may face even greater language barriers compared to Latinos. Future research should expand this study and examine barriers to SAVAME service provision for other immigrant groups.

Previous studies with Latino immigrant samples have documented the high burden of mental health and interpersonal violence among this population [30, 31]. In general, lack of access to mental health and substance use services and barriers identified by providers in our study are consistent with previous research on Latino and immigrants’ access to health and behavioral services [27, 32, 33]. A 2019 national survey of Hispanic individuals indicated that about 92% of those with a substance use disorder and 66% of those with a mental health illness did not receive any treatment in the previous 12 months [34].

Findings from this study stress the need for integrated funding streams in order to promote a syndemic approach in the prevention and treatment of SAVAME syndemic issues in vulnerable populations. This funding could not only help with increasing the availability of SAVAME services, but also support the hiring of Latino providers and the provision of training to increase cultural competency and trauma-informed care for staff at Latino-serving organizations. Similarly, these funds could support efforts to increase organizational capacity to conduct continued community education and person-to-person outreach within local immigrant communities, which is paramount to increase awareness and trust on the network of services among Latino immigrants.

Latino-serving organizations providing SAVAME services should adopt routine screening for all four syndemic factors and strive to connect their Latino immigrant clients to other SAVAME services, ideally within their organization, or elsewhere in the community. Routine screening and “warm” referrals are critical to reduce the burden of this syndemic issues among Latino immigrants and could help increase the effectiveness of the services offered for each individual SAVAME factor. For example, research has documented that for Latino immigrants with HIV, unaddressed trauma and substance use are among the chief barriers to retention into HIV care [35]. Screening HIV + Latino clients for mental health and substance use and linkage to appropriate services to address these issues has the potential to increase retention in HIV care and better HIV outcomes in this population.

Professionals at Latino-serving organizations can serve as critical gateways to connect Latino immigrants to unmet SAVAME services but, in order to do so, they need to be abreast of available, trustworthy services in an always evolving service network. Interventions to increase providers’ awareness about adequate and accessible services for their Latino immigrant clients could aid with this objective. Initiatives to foster interorganizational connections and partnerships between Latino-serving organizations could increase knowledge, facilitate “warm” referrals, and help reduce barriers to service utilization [36]. Provider awareness and interorganizational collaborations could be cultivated through communitywide coalitions of Latino-serving organizations led by independent and trustworthy brokers. In the initial months of the COVID-19 pandemic, and based in part on this formative work, our community academic team spearheaded the formation of the Latino Health Collective, a coalition of over 30 Latino-serving organizations, moderated by a well-trusted, independent academic broker. This coalition held regular town halls attended by representatives from these organizations, the local department of public health, the city’s office of immigrant affairs, and Latino-immigrant community members to identify the most pressing needs of Latino immigrant communities during the pandemic, leverage and share information about existing resources, and promote adoption of culturally-tailored testing and vaccination strategies to reduce COVID-19 disparities for this group. This initiative, currently under evaluation, holds much promise for increasing the awareness, accessibility, and adequacy of services related to other health issues, including SAVAME.

Community-based programs must also be implemented to increase knowledge of and trust in existing SAVAME services among members of the Latino immigrant community and to reduce stigma and fear of seeking services in this population. A Spanish-language, centralized, and continuously updated online repository of SAVAME services for Latino immigrants could help both Latino immigrant community members and Latino-serving providers. Similarly, peer-driven interventions have been found to increase access to health care and help reduce health disparities among hard-to-reach, marginalized communities [37–39]. The Popular Opinion Leader (POL) model is a peer-driven approach based on Diffusion of Innovation Theory [39]. POL programs recruit and train influential community members to serve as early adopters and promoters of knowledge about services and new behavioral norms (e.g., risk reduction practices, utilization of services, etc.) within their social networks [40, 41]. Peers also play a critical role disseminating health information, connecting community members to services and providing feedback to organizations for service improvement [42]. Peer-based approaches have been used successfully to address health disparities among Latinos and other ethnic/racial minorities [43, 44]. The authors are currently testing the effectiveness of these approaches to increase access to SAVAME services among Latino immigrants. Support for community-engaged research to build from, and evaluate, these home-grown programs and interventions is urgently needed.

This study presents several limitations. Although generalizability is not the goal of qualitative methods, it is important to bear in mind that the views of providers in our study may not reflect what other staff within and outside these organizations think regarding SAVAME services for Latino immigrants. Similarly, the findings may not be generalizable to other U.S. locales with different levels of urbanicity, demographics, migration history, local politics, or immigration and healthcare policies. The consistency of findings from this study, the LINKS survey [20, 21], and previous research examining the perspectives of service providers on barriers faced by Latino immigrants in other parts of the U.S. [27–29] adds external validity to our findings. Providers’ responses could also have been biased due to social desirability or out of caution. Assurances of confidentiality during the informed consent process and other procedures to increase the privacy of the interviews should have reduced this bias. Latino immigrants are not a monolithic group. Our findings reflect the importance of immigration status, health insurance, language and income, but there may be other important variables that render some Latino groups more likely to struggle to find and access SAVAME services than others. Future research must advance a more nuanced understanding of intragroup differences in availability and access of SAVAME services for Latino immigrants.

In summary, Latino-serving providers perceive important limitations in the availability, accessibility, and adequacy of SAVAME services for Latino immigrants. The challenges to meet the needs of this group are especially acute for mental health and substance use disorders, partly because of insufficient funding streams to support these services. Lack of health insurance, immigration status, LEP, stigma surrounding SAVAME issues, and limited knowledge of available services among providers and community members are the main barriers preventing access to services. Limited cultural competency, insufficient consideration of trauma, and scarcity of Latino immigrant providers results lower the quality of SAVAME services for Latino immigrant clients. Overall, small size and low levels of infrastructure of most Latino-serving organizations limits the ability to adopt a syndemic approach in the prevention and treatment of SAVAME services. Communitywide coalitions and other strategies to connect and foster collaboration across SAVAME providers working with Latino immigrant clients could improve some of these barriers and strengthen the network of Latino-serving organizations.

## Supplementary Information

**Additional file 1.** 

**Additional file 2.** 

## Data Availability

Interview transcripts cannot be shared openly due to confidentiality issues. They can however be made available to interested researchers on a case-by-case basis, upon request to the first author, Ana Martinez-Donate (apm78@drexel.edu), and contingent on obtaining IRB approval from the researchers’ institutional IRB and/or establishment of a reliance agreement between their institutional and Drexel University’s IRB.
