# Supplementary material for: Provider perceptions of availability, accessibility, and adequacy of health and behavioral services for Latino immigrants in Philadelphia: a qualitative study
Source: BMC Public Health. 2022 Aug 30;22:1645. doi: 10.1186/s12889-022-14066-z (PMC9427076; doi:10.1186/s12889-022-14066-z)
Supplement: Supplementary file 1 — Additional file 1. [file 12889_2022_14066_MOESM1_ESM.pdf]

EXPLORATORY/DEVELOPMENT GRANT **Federal Award Date:** 02/28/2019
Department of Health and Human Services
National Institutes of Health

NATIONAL INSTITUTE ON MINORITY HEALTH AND HEALTH DISPARITIES

Grant Number: 1R21MD012352-01A1
FAIN: R21MD012352

Principal Investigator(s):
Ana P Martinez-Donate, PHD

Project Title: CRISOL: Building Community Resilience and Integrating Efforts to Understand and Address Syndemic Health Conditions Afflicting Young LatinoImmigrants

Mr. Sullivan, Robert
Assistant Director, Pre-Award Administration
1505 Race St, 10th Floor
OFFICE OF RESEARCH
PHILADELPHIA, PA 191021119

Award e-mailed to: scl38@drexel.edu

Period Of Performance:
Budget Period: 03/01/2019 – 11/30/2019
Project Period: 03/01/2019 – 11/30/2020

Dear Business Official:

The National Institutes of Health hereby awards a grant in the amount of $198,755 (see “Award Calculation” in Section I and “Terms and Conditions” in Section III) to DREXEL UNIVERSITY in support of the above referenced project. This award is pursuant to the authority of 42 USC 241 42 CFR 52 and is subject to the requirements of this statute and regulation and of other referenced, incorporated or attached terms and conditions.

Acceptance of this award including the “Terms and Conditions” is acknowledged by the grantee when funds are drawn down or otherwise obtained from the grant payment system.

Each publication, press release, or other document about research supported by an NIH award must include an acknowledgment of NIH award support and a disclaimer such as “Research reported in this publication was supported by the National Institute On Minority Health And Health Disparities of the National Institutes of Health under Award Number R21MD012352. The content is solely the responsibility of the authors and does not necessarily represent the official views of the National Institutes of Health.” Prior to issuing a press release concerning the outcome of this research, please notify the NIH awarding IC in advance to allow for coordination.

Award recipients must promote objectivity in research by establishing standards that provide a reasonable expectation that the design, conduct and reporting of research funded under NIH awards will be free from bias resulting from an Investigator’s Financial Conflict of Interest (FCOI), in accordance with the 2011 revised regulation at 42 CFR Part 50 Subpart F. The Institution shall submit all FCOI reports to the NIH through the eRA Commons FCOI Module. The regulation does not apply to Phase I Small Business Innovative Research (SBIR) and Small Business Technology Transfer (STTR) awards. Consult the NIH website http://grants.nih.gov/grants/policy/coi/ for a link to the regulation and additional important information.

If you have any questions about this award, please contact the individual(s) referenced in Section IV.

Sincerely yours,

Priscilla Grant
Grants Management Officer
NATIONAL INSTITUTE ON MINORITY HEALTH AND HEALTH DISPARITIES

Additional information follows

SECTION I – AWARD DATA – 1R21MD012352-01A1

Award Calculation (U.S. Dollars)

**Federal Direct Costs**	$127,000
**Federal F&A Costs**	$71,755
**Approved Budget**	$198,755
**Total Amount of Federal Funds Obligated (Federal Share)**	$198,755
**TOTAL FEDERAL AWARD AMOUNT**	$198,755
**AMOUNT OF THIS ACTION (FEDERAL SHARE)**	$198,755

SUMMARY TOTALS FOR ALL YEARS		
YR	THIS AWARD	CUMULATIVE TOTALS
1	$198,755	$198,755
2	$227,145	$227,145

Recommended future year total cost support, subject to the availability of funds and satisfactory progress of the project

Fiscal Information:
CFDA Name: Minority Health and Health Disparities Research
CFDA Number: 93.307
EIN: 1231352630A1
Document Number: RMD012352A
PMS Account Type: P (Subaccount)
Fiscal Year: 2019

IC	CAN	2019	2020
MD	8472687	$198,755	$227,145

Recommended future year total cost support, subject to the availability of funds and satisfactory progress of the project

NIH Administrative Data:
PCC: CPS01NJ / **OC**: 414A / **Released**: GRANTP 02/25/2019
Award Processed: 02/28/2019 12:11:41 AM

SECTION II – PAYMENT/HOTLINE INFORMATION – 1R21MD012352-01A1

For payment and HHS Office of Inspector General Hotline information, see the NIH Home Page at http://grants.nih.gov/grants/policy/awardconditions.htm

SECTION III – TERMS AND CONDITIONS – 1R21MD012352-01A1

This award is based on the application submitted to, and as approved by, NIH on the above-titled project and is subject to the terms and conditions incorporated either directly or by reference in the following:

a. The grant program legislation and program regulation cited in this Notice of Award.
b. Conditions on activities and expenditure of funds in other statutory requirements, such as those included in appropriations acts.
c. 45 CFR Part 75.
d. National Policy Requirements and all other requirements described in the NIH Grants Policy Statement, including addenda in effect as of the beginning date of the budget period.
e. Federal Award Performance Goals: As required by the periodic report in the RPPR or in the final progress report when applicable.
f. This award notice, INCLUDING THE TERMS AND CONDITIONS CITED BELOW.

(See NIH Home Page at http://grants.nih.gov/grants/policy/awardconditions.htm for certain references cited above.)

Research and Development (R&D): All awards issued by the National Institutes of Health (NIH) meet the definition of "Research and Development" at 45 CFR Part§ 75.2**.** As such, auditees should identify NIH awards as part of the R&D cluster on the Schedule of Expenditures of Federal Awards (SEFA). The auditor should test NIH awards for compliance as instructed in Part V, Clusters of Programs. NIH recognizes that some awards may have another classification for purposes of indirect costs. The auditor is not required to report the disconnect (i.e., the award is classified as R&D for Federal Audit Requirement purposes but non-research for indirect cost rate purposes), unless the auditee is charging indirect costs at a rate other than the rate(s) specified in the award document(s).

An unobligated balance may be carried over into the next budget period without Grants Management Officer prior approval.

This grant is subject to Streamlined Noncompeting Award Procedures (SNAP).

This award is subject to the requirements of 2 CFR Part 25 for institutions to receive a Dun & Bradstreet Universal Numbering System (DUNS) number and maintain an active registration in the System for Award Management (SAM). Should a consortium/subaward be issued under this award, a DUNS requirement must be included. See http://grants.nih.gov/grants/policy/awardconditions.htm for the full NIH award term implementing this requirement and other additional information.

This award has been assigned the Federal Award Identification Number (FAIN) R21MD012352. Recipients must document the assigned FAIN on each consortium/subaward issued under this award.

Based on the project period start date of this project, this award is likely subject to the Transparency Act subaward and executive compensation reporting requirement of 2 CFR Part 170. There are conditions that may exclude this award; see http://grants.nih.gov/grants/policy/awardconditions.htm for additional award applicability information.

In accordance with P.L. 110-161, compliance with the NIH Public Access Policy is now mandatory. For more information, see NOT-OD-08-033 and the Public Access website: http://publicaccess.nih.gov/.

In accordance with the regulatory requirements provided at 45 CFR 75.113 and Appendix XII to 45 CFR Part 75, recipients that have currently active Federal grants, cooperative agreements, and procurement contracts with cumulative total value greater than $10,000,000 must report and maintain information in the System for Award Management (SAM) about civil, criminal, and administrative proceedings in connection with the award or performance of a Federal award that reached final disposition within the most recent five-year period. The recipient must also make semiannual disclosures regarding such proceedings. Proceedings information will be made publicly available in the designated integrity and performance system (currently the Federal Awardee Performance and Integrity Information System (FAPIIS)). Full reporting requirements and procedures are found in Appendix XII to 45 CFR Part 75. This term does not apply to NIH fellowships.

Treatment of Program Income:
Additional Costs

SECTION IV – MD Special Terms and Conditions – 1R21MD012352-01A1

Clinical Trial Indicator: No
This award does not support any NIH-defined Clinical Trials. See the NIH Grants Policy Statement Section 1.2 for NIH definition of Clinical Trial.

RESTRICTION: This award is issued without a currently valid certification of IRB approval for the second phase of this project (the survey design phase) with the following special condition: Only activities that are clearly severable and independent from activities that involve human subjects in the second phase of this project may be conducted under this award until the project has received IRB approval consistent with 45 CFR Part 46 and certification of IRB approval has been submitted to and accepted by the NIMHD.

No funds may be drawn down from the payment system and no obligations may be made against Federal funds for research involving human subjects at any site engaged in the second phase of such research for any period not covered by both (1) the awardee's OHRP-approved Assurance and if performance sites are involved, each performance site's OHRP-approved Assurance(s) and (2) appropriate IRB approvals consistent with all OHRP-approved Assurances.

Failure to comply with this special condition can result in the suspension and/or termination of this award, withholding of support, audit disallowances, and/or other appropriate action.

REQUIREMENT: This award is subject to the conditions set forth in PAR-18-285, Addressing Health Disparities through Effective Interventions Among Immigrant Populations (R21 Clinical Trial Optional), NIH Guide to Grants and Contracts, 11/01/2017, which is hereby incorporated by reference as special terms and conditions of this award.

Copies of this RFA may be accessed at the following internet address: http://www.nih.gov/grants/guide/index.html

Copies may also be obtained from the Grants Management Contact indicated in the terms of award.

REQUIREMENT: Use of humans and animals in any new activities must be requested prior to the start of the activity and must be approved in writing in advance by the NIMHD. See NOT-MD-08-002, "Guidance and Clarification on NCMHD Policy on Prior Approval for Subprojects and Pilot Projects Involving Human Subjects or Vertebrate Animals," NIH Guide to Grants and Contracts, April 29, 2008, which is hereby incorporated by reference as special terms and conditions of this award. See also NOT-OD-15-129, "Prior NIH Approval of Human Subjects Research in Active Awards Initially Submitted without Definitive Plans for Human Subjects Involvement (Delayed Onset Awards): Updated Notice," and NIH-OD-15-128, "Guidance on Changes That Involve Human Subjects in Active Awards and That Will Require Prior NIH Approval: Updated Notice."

Copies of these Notices may be accessed at the following internet address: http://www.nih.gov/grants/guide/index.html

Copies may also be obtained from the Grants Management Contact indicated in the terms of award.

RESTRICTION: Stipends and payments made for educational assistance (e.g., scholarships, fellowships, and student aid costs) may not be paid from NIH research grant funds even when they would appear to benefit the research project (NIH GPS Section 7.9.1). Compensation must be in accordance with organizational policies consistently applied to both federally and non-federally supported activities and must be supported by acceptable accounting records that reflect the employer-employee relationship. Under these conditions, the funds provided as compensation for services rendered are not considered stipend supplementation; they are allowable charges to Federal grants, including PHS research grants. (A stipend is a payment made to an individual under a fellowship or training grant in accordance with pre-established levels to provide for the individual's living expenses during the period of training. A stipend is not considered compensation for the services expected of an employee.) See the NIH Grants Policy Statement for allowable forms of student compensation, available at http://grants.nih.gov/grants/policy/nihgps/nihgps.pdf

INFORMATION: In order to redistribute awards more evenly throughout the year, budget periods are being adjusted. This award is issued with a 9-month budget period and with 12 months of support. Continuation awards will cycle each year on December 1st.

INFORMATION: Although the budget period start date for this award is March 1st, this award includes funds for 12 months of support. Future year budget periods will cycle on December 1st. Allowable preaward costs may be charged to this award, in accordance with the conditions outlined in the NIH Grants Policy Statement, and with institutional requirements for prior approval. The NIH GPS can be found on the internet at http://grants.nih.gov/grants/policy/nihgps/nihgps.pdf.

INFORMATION: See the Certificate of Confidentiality policy at https://humansubjects.nih.gov/coc/major-changes. This policy protects against the involuntary release of personally identified research information of a sensitive nature sought through any federal, state, or local civil, criminal, administrative, legislative, or other proceedings.

INFORMATION: This award reflects the NIMHD's acceptance of the certification that all key personnel have completed education on the protection of human subjects, in accordance with NIH policy, "Required Education in the Protection of Human Research Participants," as announced in the June 5, 2000 NIH Guide (revised August 25, 2000) (http://grants.nih.gov/grants/guide/notice-files/NOT-OD-00-039.html).

Any individual involved in the design and conduct of the study that is not included in the certification must satisfy this requirement prior to participating in the project. Failure to comply can result in the suspension and/or termination of this award, withholding of support of the continuation award, audit disallowances, and/or other appropriate action.

INFORMATION: See "Federalwide Assurance Requirements" and "Certification of IRB Approval" under the Human Subjects Protections section in the NIH Grants Policy Statement (NIHGPS), for specific requirements and recipient responsibilities related to the protection of human subjects, which are applicable to and are a term and condition of this award. The NIHGPS can found on the internet at http://grants.nih.gov/grants/policy/nihgps/nihgps.pdf

INFORMATION: This is a Modular Grant Award without direct cost categorical breakdowns issued in accordance with the guidelines published in the NIH Grants Policy Statement, see http://grants.nih.gov/grants/policy/nihgps/nihgps.pdf.htm
Recipients are required to allocate and account for costs related to this award by category within their institutional accounting system in accordance with applicable cost principles.

INFORMATION: None of the funds in this award shall be used to pay the salary of an individual at a rate in excess of the current salary cap. See the new Salary Limitations on Grants: https://grants.nih.gov/grants/guide/notice-files/NOT-OD-18-181.html

INFORMATION: Unobligated balances may be used by the NIMHD to reduce or offset funding for a subsequent budget period.

INFORMATION: Regarding changes in scope, attention is called to the NIH Grants Policy Statement. The Change in Scope section is found in Section 8.1.2 at http://grants.nih.gov/grants/policy/nihgps/nihgps.pdf. The recipient must obtain prior approval from the NIMHD for a change in the direction, aims, objectives, purposes, or type of research or training, or other areas that constitute a significant change in the approved project. Specific examples are provided.

INFORMATION: Regarding allowability of selected items of cost, attention is called to the NIH Grants Policy Statement. The Selected Items of Cost section is found in Section 7.9.1 at http://grants.nih.gov/grants/policy/nihgps/nihgps.pdf

INFORMATION: Honoraria are unallowable when the primary intent is to confer distinction on, or to symbolize respect, esteem, or admiration for, the recipient of the honorarium. A payment for services rendered, such as a speaker's fee under a conference grant, is allowable. See Section 7.9.1 at http://grants.nih.gov/grants/policy/nihgps/nihgps.pdf

INFORMATION: This award includes funds awarded for consortium activity. Consortia are to be established and administered as described in the NIH Grants Policy Statement (NIH GPS). The referenced section of the NIH GPS is available at: http://grants.nih.gov/grants/policy/nihgps/nihgps.pdf. See "Consortium Agreements" in Section 15 for specific responsibilities and requirements for recipients and consortium participants, which are applicable to and are a term and condition of this award.

INFORMATION: For administrative and management concerns, contact the Grants Management Specialist, Sy L. Shackleford, at (301) 451-8542. For programmatic and scientific concerns, contact the Program Director, Dr. Nancy Lynne Jones, at (301) 594-8945.

STAFF CONTACTS

The Grants Management Specialist is responsible for the negotiation, award and administration of this project and for interpretation of Grants Administration policies and provisions. The Program Official is responsible for the scientific, programmatic and technical aspects of this project. These individuals work together in overall project administration. Prior approval requests (signed by an Authorized Organizational Representative) should be submitted in writing to the Grants Management Specialist. Requests may be made via e-mail.

Grants Management Specialist: Sy Shackleford
Email: shacklefords@mail.nih.gov **Phone**: 301-402-1366

Program Official: Nancy Lynne Jones
Email: jonesna@mail.nih.gov **Phone**: 301-594-8945

SPREADSHEET SUMMARY
GRANT NUMBER: 1R21MD012352-01A1

INSTITUTION: DREXEL UNIVERSITY

Facilities and Administrative Costs	Year 1	Year 2
F&A Cost Rate 1	56.5%	56.5%
F&A Cost Base 1	$127,000	$133,000
F&A Costs 1	$71,755	$75,145
