# Supplementary material for: Provider perceptions of availability, accessibility, and adequacy of health and behavioral services for Latino immigrants in Philadelphia: a qualitative study
Source: BMC Public Health. 2022 Aug 30;22:1645. doi: 10.1186/s12889-022-14066-z (PMC9427076; doi:10.1186/s12889-022-14066-z)
Supplement: Supplementary file 2 — Additional file 2. [file 12889_2022_14066_MOESM2_ESM.pdf]

DREXEL UNIVERSITY Office of Research

Office of Research
Human Research Protection Program
Drexel University
Bellet Building
1505 Race Street, 7th Floor
Philadelphia, PA 19102

Institutional Review Board
Phone: (215) 762-3944
Fax: (215) 762-6258
E-mail: HRPP@drexel.edu

Association for the Accreditation of Human Research Protection Programs, Inc. Full Accreditation

Approval for a Project Involving Human Subjects Research that Does Not Require Continuing Review

Date: May 30, 2019

Protocol Number:	1810006698
PI:	Ana P. Martinez-Donate
Review Type:	Expedited
Review Date:	May 30, 2019
Approved On:	May 30, 2019
Committee:	IRB 3
Sponsor:	National Institute of Health
Project Title:	Understanding Latino Immigrant Syndemic Conditions: LINKS2

The IRB determined that the research meets the approval criteria set forth in: 45 CFR 46.111, 21 CFR 56.111. The study was approved under Expedited review.

It was also determined that the research **does not require a continuing review**. Consequently, there is no IRB approval period listed for this project.

If applicable to your study, you can access your IRB-approved, stamped consent document or consent script through Coeus. Open the Attachments tab to find the approval letter or approval packet. The stamped documents are labeled as such. **Copies of the IRB approved stamped consent document or consent script must be used in obtaining consent.**

Please note: All applicable Institutional and non-Institutional approvals must also be secured prior to study implementation. These approvals include, but are not limited to, Radiation Safety Committee (“RSC”); Institutional Biosafety Committee ("IBC"); and Conflict of Interest, (“COI”); individual departmental and external site approvals.

Finally, in conducting this research, you are obligated to submit the following:

- **Amendment requests - All changes to the research must be reviewed and approved by the IRB.** Changes requiring approval include, but are not limited to, changes in the design or focus of the research project, revisions to the information sheet for participants, addition of new measures or instruments, increasing the subject number, and changes to the research funding. **Changes made to eliminate apparent immediate hazards to subjects and implemented prior to IRB approval must be promptly reported to theIRB.**

- **Reportable New Information** – Using the Reportable New Information e-form, report new information items such as those described in HRP-214 Form - Reportable New Information to the IRB **within 5 days**.

- **Final Report (Closure report)** - Submit when the study is permanently closed to enrollment, all subjects have completed all protocol related interventions and interactions, collection of private identifiable information is complete, and analysis of private identifiable information is complete. If the Principal Investigator is leaving Drexel, the study must either be formally closed or a Modification must be submitted to approve a new Principal Investigator.

For the complete list of investigator responsibilities, please see the Investigator Manual (HRP-103) and other Policies and Procedures found on the Drexel University IRB website.

Please contact the IRB at (215) 762-3944 or HRPP@drexel.edu if you have any questions.

Drexel University - Project CRiSOL 2
Consent for Key Informant Interviews (KIIs)

1. Title of research study:

Understanding Latino ImmigraNt Syndemic Conditions **(LINkS 2)**

2. Researcher: Ana Martinez-Donate, PhD

3. Concise Summary of Key Information:

This study seeks to better understand the prevalence and determinants of four health factors, HIV/AIDS, Substance Use, Violence, and Mental Health, also known as "SAVAME," among Latinos in Philadelphia and to identify strategies to better address these factors. Your consent is being sought for research and the participation is voluntary. During the study, you will answer questions about a specific topic. You may ask questions and talk about topics you feel are important and relevant to the questions asked of you. Your opinion matters. The length of your interview is approximately 45 minutes on one day. The interview will be conducted by a research assistant from Drexel University. The interview will be completed at a mutually agreed location such as your organization, Drexel University, the Philadelphia AIDS Consortium (TPAC), or a location that is private and confidential (such as a meeting room in a public library). You may receive some benefits from participating in this research, but there is no guarantee. Participating in this study will expose you to very minimal risk. You are not required to take part in this study. It is your choice to participate. You may decide if you want to consent, withdraw, or decline at any time. You will be given a $20 gift card incentive to participate in this study, and it will not cost you anything to participate.

4. Why you are being invited to take part in a research study

We invite you to take part in a research study because you have indicated that you are a leader or provider at an organization in Philadelphia that offers health, legal, educational, spiritual, and/or social services to Latino immigrants in Philadelphia. Please listen carefully and ask any questions you may have before agreeing to take part in the study.

5. What you should know about a research study

If you agree to be in this study, we will conduct an audio-recorded interview with you. The interview will include questions about the following:

- Your organization and the services your organization provides
- Your opinion about the service delivery received by your organization's clientele
- Your opinion of the impact HIV/AIDS, Substance Use, Violence, and Mental Health in Philadelphia
- Your opinion of the strengths and weaknesses of all services for Latino immigrants in Philadelphia
- Recommendations to improve the health of Latino immigrants in Philadelphia related to HIV/AIDS, Substance Use, Violence, and Mental Health.

The interview will take about 1 hour to complete.

APPROVED
Human Research Protection
Protocol # 1810006698
Approval Date: 05/30/2019

- Someone will explain this research study to you.
- Whether or not you take part is up to you.
- You can choose not to take part.
- You can agree to take part now and you can change your mind later.
- If you decide to not be a part of this research no one will hold it against you.
- Feel free to ask all the questions you want before you decide.

6. Who can you talk to about this research study?

If you have questions, concerns, or complaints, or think the research has hurt you, talk to the Principal Investigator Dr. Ana P. Martinez-Donate, Associate Professor at Drexel University Dornsife School of Public Health. You may contact her by phone at 267-359-6124 or by email at apm78@drexel.edu. Please ask any questions you may have now. If you have any further questions or concerns regarding your rights as a subject in this study, you may contact the Institutional Review Board (IRB).

This research has been reviewed and approved by an Institutional Review Board (IRB). An IRB reviews research projects so that steps are taken to protect the rights and welfare of human subjects taking part in research. You may talk to them at (215) 762-3944 or email HRPP@drexel.edu for any of the following:

- Your questions, concerns, or complaints are not being answered by the research team.
- You cannot reach the research team.
- You want to talk to someone besides the research team.
- You have questions about your rights as a research subject.
- You want to get information or provide input about this research.

7. Why are we doing this research?

In the United States, the largest racial/ethnic minority consists of Latino individuals; currently many social issues increase the frequency and/or worsen the impact of certain health issues on Latino immigrants. One of these issues is the limited access to health care many Latinos have. The purpose of this study is to learn how service providers perceive the accessibility, availability, and adequacy of health services for Latino immigrants in Philadelphia. There will be 30 interviews conducted with organizations that serve Latino immigrants in Philadelphia. Your responses regarding Latino immigrant experiences may improve health service accessibility in Philadelphia.

8. How long will the research last?

We expect that you will be in this research study for about 45 minutes on one day.

9. How many people will be studied?

We expect about 30 people in Philadelphia will participate in these "key informant interviews".

10. What happens if I say yes, I want to be in this research?

- You will earn a $20 gift card for study participation

APPROVED
Human Research Protection
Protocol # 1810006698
Approval Date: 05/30/2019

- Taking part in this study is completely voluntary. You may skip any questions that you do not want to answer. If you decide not to take part or to skip some of the questions, it will not affect your current or future relationship with Drexel University.
- If you decide to take part, you are free to withdraw at any time. You can agree to take part now, then change your mind later.
- You will be part of a 45-minute audio-recorded in-person interview with a trained member of our research team. The interview is semi-structured and you will be asked questions. You can add any additional information, even if not specifically asked by the question. If you do not feel comfortable answering questions you may refuse to do so. There are no right or wrong answers. We want to hear your ideas, opinions, and experiences.
- We will audio-record this session, then transcribe the interview after the in-depth interview takes place. You should not mention anyone's name, but if that occurs, we will delete that from our record. We will analyze the information obtained from the interview; we will not identify individuals, but only group results in our reports. The research team's results will result in presentations and papers.
- At the end of the interview we will provide you with a brief demographic survey to complete. This survey is also voluntary and you can refuse to complete it or skip any quetions. Any identifying information will be excluded.
- The interview will be completed at a mutually agreed location such as your organization, Drexel University, the Philadelphia AIDS Consortium (TPAC), or a location that is private and confidential (such as a meeting room in a public library).

11. What are my responsibilities if I take part in this research?

If you take part in this research, it is very important that you:

- Follow the investigator's or researcher's instructions.
- Tell the investigator or researcher right away if you have a complication or injury.

12. What happens if I do not want to be in this research?

You may decide not to take part in the research, and it will not be held against you. If you choose to reschedule your interview, it will not be held against you.

13. What happens if I say yes, but I change my mind later?

If you agree to take part in the research now, you can stop at any time it will not be held against you.

14. Is there any way being in this study could be bad for me?

There is the risk that you may find some of the questions about your job and health services provided to be sensitive. You can refuse participation at any time or reschedule the interview. Refusing to participate or rescheduling will not be held against you.

15. Do I have to pay for anything while I am on this study?

There is no cost to you for participating in this study.

APPROVED
Human Research Protection
Protocol # 1810006698
Approval Date: 05/30/2019

16. Will being in this study help me in any way?

We cannot promise any direct benefits to you or others from your taking part in this research. However, possible benefits include contributing to research about Latino immigrant and Latino access, availability, and adequacy of health services in Philadelphia. You may find it fulfilling to contribute to the current research regarding Latino health. This benefit may not continue after participation.

17. What happens to the information we collect?

Efforts will be made to limit your personal information. We will not collect name, address, or other identifying information. We will also restrict access to the study data to people who have a need to review this information. We cannot promise complete secrecy. Organizations that may inspect and copy your information include the IRB and other Drexel University representatives. If we create a public report about the study, we will not include any information that will make it possible to identify you. Research records, including the audio-recorded interview, will only be accessible to the study researchers via password-encrypted files. The Drexel IRB may request these files in the future, but once again, your personal information will not be shared outside of the research, unless mandated by law.

We may publish the results of this research. However, we will not collect your name or other information that may identify you.

18. Can I be removed from the research without my OK?

The person in charge of the research study or the sponsor can remove you from the research study without your approval. Possible reasons for removal include you not meeting eligibility criteria or the researcher deeming the interview too emotionally charged. We will tell you about any new information that may affect your health, welfare, or choice to stay in the research.

19. Certificate of Confidentiality

The study team has obtained a Certificate of Confidentiality to further protect your privacy. With this Certificate, the investigators may not disclose research information that may identify you in any Federal, State, or local civil, criminal, administrative, legislative, or other proceedings, for example, if there is a court subpoena. The researchers will use the Certificate to resist any demands for information that would identify you unless:

1. There is a law that requires disclosure (such as to report child abuse or communicable diseases but not for legal proceedings);
2. The research information is used for other scientific research, as allowed by federal regulations protecting research subjects.

You should understand that a Certificate of Confidentiality does not prevent you or a member of your family from voluntarily releasing information about yourself or your involvement in this research. Finally, you should understand that the investigator is not prevented from taking steps, including reporting to authorities, to prevent serious harm to yourself or others.

20. What else do I need to know?

This research study is being conducted by Drexel University and is funded by the National Institutes of Health.

APPROVED
Human Research Protection
Protocol # 1810006698
Approval Date: 05/30/2019

If you agree to take part in this research study, we will give you a $20 gift card for your time and effort.

Interviews are expected to be completed by the end of February 2020.

Your signature documents your permission to take part in this research.

Signature of subject	Date
Printed name of subject	
Signature of person obtaining consent	Date
Printed name of person obtaining consent	Form Date

My signature below documents that the information in the consent document and any other written information was accurately explained to, and apparently understood by, the subject, and that consent was freely given by the subject.

Signature of witness to consent process	Date
Printed name of person witnessing consent process	

APPROVED
Human Research Protection
Protocol # 1810006698
Approval Date: 05/30/2019
